# Supplementary material for: Earthquake focal mechanisms with distributed acoustic sensing
Source: Nat Commun. 2023 Jul 13;14:4181. doi: 10.1038/s41467-023-39639-3 (PMC10345142; doi:10.1038/s41467-023-39639-3)
Supplement: Supplementary file 1 — Supplementary Information [file 41467_2023_39639_MOESM1_ESM.pdf]

Earthquake focal mechanisms with distributed acoustic sensing

J. Li et al.,

Supplementary Information

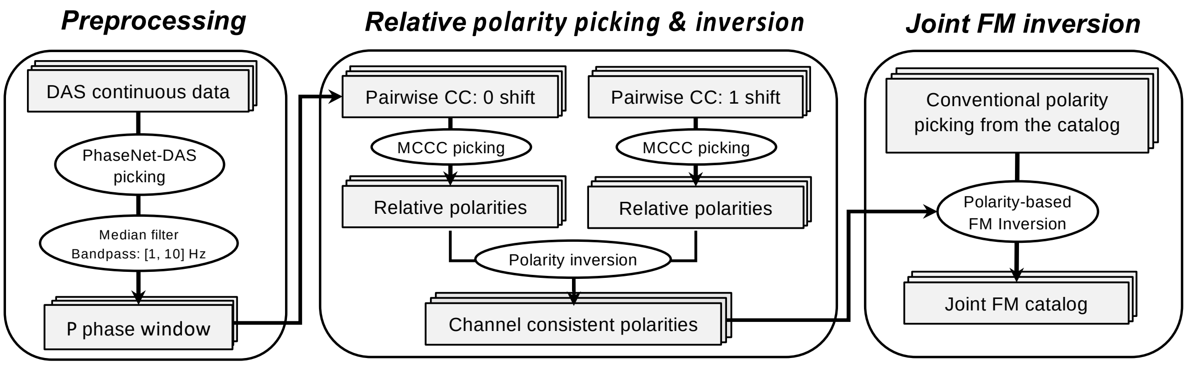

Figure S1. **Workflow of leveraging DAS for earthquake focal mechanism inversion**. The workflow consists of three steps. In the preprocessing step, we first use the PhaseNet-DAS to pick the P-phase arrival. We apply a median filter and a bandpass filter between 1 to 10 Hz and extract a two-second window along the picked P-phase arrival. In the next step, we perform cross-correlations between all earthquake pairs in a cluster, both at the same channel and at adjacent channels. We use the MCCC to pick the relative polarities and derive the channel-consistent polarity. Finally, we input both DAS and conventional polarity picks to search for a jointly inverted focal mechanism.
